# Serum Sclerostin as a Possible Biomarker in Ankylosing Spondylitis: A Case-Control Study

**DOI:** 10.1155/2018/9101964

**Published:** 2018-05-02

**Authors:** Fabio Massimo Perrotta, Fulvia Ceccarelli, Cristiana Barbati, Tania Colasanti, Antonia De Socio, Silvia Scriffignano, Cristiano Alessandri, Ennio Lubrano

**Affiliations:** ^1^Academic Rheumatology Unit, Dipartimento di Medicina e Scienze della Salute, Università degli studi del Molise, Campobasso, Italy; ^2^Dipartimento di Medicina Interna e Specialità Mediche-UOC di Reumatologia-“Sapienza”, Università di Roma, Roma, Italy

## Abstract

**Objective:**

Several molecules are involved in the pathogenesis of a new bone formation in ankylosing spondylitis (AS). The aim of this study was to evaluate the serum levels of sclerostin in patients with AS as a possible biomarker and to investigate any correlations with radiographic damage, disease activity, and function.

**Methods:**

AS patients fulfilled the modified New York criteria, and healthy controls were enrolled for this study. BASDAI, ASDAS-CRP, BASMI, BASFI, patient and physician VAS, and C-reactive protein were evaluated at baseline visit. Spinal damage was assessed using the mSASSS on radiographs performed within 3 months from baseline. Serum concentrations of sclerostin were assessed at baseline and after four months of therapy in patients who started an anti-TNF.

**Results:**

Twenty healthy subjects and 40 AS patients were enrolled in the study. In our group, serum sclerostin levels (median (25th–75th percentile)) were significantly higher in healthy controls (18.04 (13.6–24) pg/ml) than in AS patients (6.46 (4.5–11.1) pg/ml; *P* value < 0.01). However, no significant correlations were found between serum sclerostin levels and radiographic damage, assessed by mSASSS, and between serum sclerostin levels and clinical indices of activity and disability or with laboratory parameters. Sclerostin levels did not show significant changes after 4 months of anti-TNF therapy.

**Conclusions:**

The results of our study suggest a possible role of sclerostin in the identification of AS patients. Further studies are needed to prove the role of sclerostin as a disease activity biomarker and progression of disease in AS.

## 1. Introduction

Ankylosing spondylitis (AS) is a chronic inflammatory disease characterized by inflammation and new bone formation at axial and peripheral entheseal sites [1]. During the disease course, the development of syndesmophytes, enthesophytes, and spinal fusion is associated with chronic pain, functional impairment, and disability [1]. The introduction of biological therapies such as the inhibitors of tumor necrosis factor-alpha (TNF) and interleukin-17 has dramatically improved the overall outcome for patients with AS and nonradiographic axial spondyloarthritis [2–4] and shifted the focus of physicians towards the prevention of structural damage in the spine and other sites, to avoid loss of function and disability.

Several mechanisms involving cellular elements (i.e., osteocytes, chondrocytes, and immune cells), inflammatory cytokines, and cellular pathways seem to be responsible for new bone formation in AS. However, the precise mechanism in which inflammation and new bone formation are coupling is still not fully understood. Furthermore, the role of biologic treatments in the inhibition of radiographic progression is still almost unclear, even in the early stage of the disease [5–8]. Recently, the role of the Wnt/*β*-catenin pathway and its inhibitors, Dikkopf and sclerostin, has been evaluated in AS pathogenesis to identify a possible link with bone formation [9–11]. Wnt proteins bind to a receptor/coreceptor complex on the plasma membrane, which consists of LRP5/6 and Frizzled proteins. The engagement of this receptor complex by Wnt proteins leads to phosphorylation of *β*-catenin that translocates in the nucleus where it is involved in the transcription of genes responsible for osteoblast differentiation and bone formation [12–14].

On these basis, it has been hypothesized that impaired expression of Wnt proteins or their inhibitors could contribute to the pathogenesis of AS. The study conducted by Appel et al. demonstrated the reduction of tissue expression of sclerostin at entheseal sites and in blood samples of AS patients compared with healthy subjects and patients affected by rheumatoid arthritis [9, 10]. Similarly, a reduction in serum levels of sclerostin and Dikkopf1 has been demonstrated in AS patients [5]. Thus, the molecules involved in Wnt/*β*-catenin could be possible useful biomarkers in AS.

The aim of this study was to evaluate the serum levels of sclerostin in patients with AS and to investigate any correlations with radiographic damage, disease activity, and function. Furthermore, a secondary aim was to evaluate the modifications of serum levels after treatment with anti-TNF agents.

## 2. Materials and Methods

The study was designed as a case-control study with a longitudinal part.

Patients affected by AS, referring to the Division of Rheumatology—Sapienza, University of Rome, and the Academic Rheumatology Unit, University of Molise, were consecutively enrolled in this study. Patients were classified according to the New York criteria [15]. Healthy subjects, matched by age and sex, were enrolled as a control group. Exclusion criteria were (1) age ≤ 18 years, (2) the presence of history of bone fractures in the previous 24 months, and (3) no treatment with bisphosphonate agents.

For all the patients enrolled in the present analysis, the following data were collected: demographic data, disease duration, extra-articular manifestations (EAM) (uveitis, inflammatory bowel diseases (IBD), and psoriasis), and clinical pattern (presence of peripheral arthritis, enthesitis, and dactylitis). All patients underwent a clinical assessment, and the following indices were evaluated:

- Swollen/tender joint count on 66 and 68 joints, respectively
- Bath Ankylosing Spondylitis Metrology Index (BASMI) [16]
- Bath Ankylosing Spondylitis Disease Activity Index (BASDAI) [17]
- Bath Ankylosing Spondylitis Functional Index (BASFI) [18]
- Patient's visual analog scale (VAS) on global disease activity spinal pain (0–100 mm) [19]

The values of erythrocyte sedimentation rate (ESR, Westergren method, mm/h) and C-reactive protein (CRP, mg/l) were registered. The Ankylosing Spondylitis Disease Activity Score (ASDAS) was also calculated [20].

Radiographs of the spine and pelvis performed within three months from the enrollment in the study were collected in all patients. Each radiograph was evaluated by an expert reader (FMP) using the modified Stoke Ankylosing Spondylitis Spinal Score (mSASSS) [21]. According to the criteria of New York, the involvement of the sacroiliac joints was evaluated by assigning a score ranging from I (doubtful) to IV (complete ankyloses) [15].

### 2.1. Determination of Sclerostin Serum Levels

Serum samples of patients and controls were collected during the visits. Serum concentrations of sclerostin was assessed using commercial kit ELISA (*AUROGENE srl, Rome, Italy*). The serum samples of patients were taken at the time of the visit and stored at −80°.

AS patients, naïve to biologic treatment who started biologic therapy (anti-TNF) due to high disease activity, were longitudinally followed up for four months. In this subgroup of patients, sclerostin serum concentrations were evaluated at baseline (sample taken the day of starting anti-TNF therapy) and after four months of anti-TNF therapy.

### 2.2. Statistical Analysis

Statistical analysis was performed using the PRISM program 5—GraphPad. Normally distributed variables were summarized using the mean ± standard deviation (SD) and nonnormally distributed variables by the median/25th–75th percentile. Percentages were used when appropriate. Mann–Whitney test was performed for unpaired categorical data and *t*-test for paired samples. Univariate comparisons between nominal variables were calculated using chi-square test or Fisher's test where appropriate. The significance of the correlation was assessed by the correlation coefficient of Spearman's rank. Two-tailed *P* values were reported. *P* values less than 0.05 were considered significant.

## 3. Results

**Figure 1:**
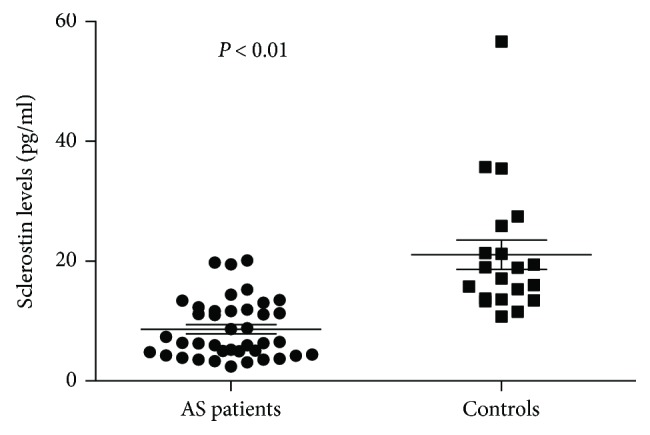
Sclerostin serum levels in patients with AS (*n* = 40) and in healthy controls (*n* = 20).

**Figure 2:**
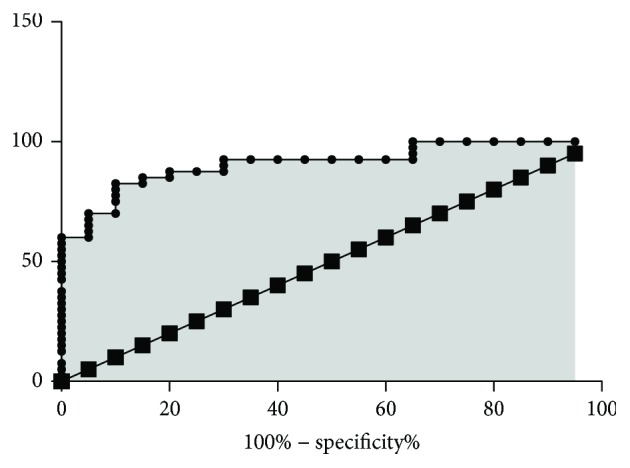
Receiver operating characteristic (ROC) curve for serum levels of sclerostin in patients with AS (*N* = 40) and in healthy controls (*n* = 20). Area under the curve: 0.91; 95% confidence interval: 0.8377 to 0.9823; *P* value: <0.01.

**Figure 3:**
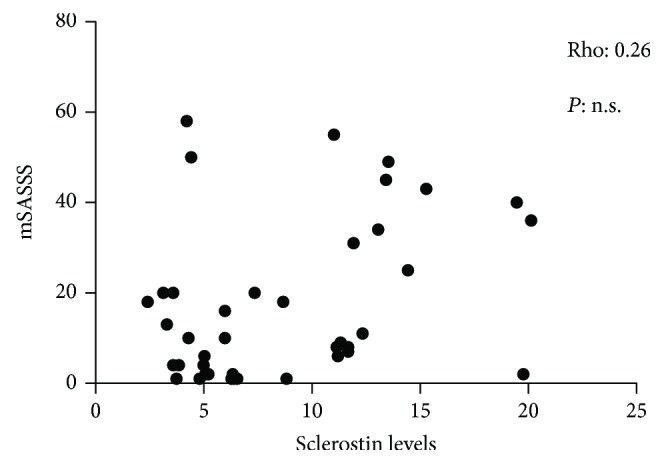
Correlation (Spearman's rho) between serum levels of sclerostin and mSASSS in patients with AS. mSASSS: modified Stoke Ankylosing Spondylitis Spinal Score.

During one-year period, we enrolled 40 AS patients and 20 age- and sex-matched controls. Of the 40 AS patients, 15 subjects who started anti-TNF treatment were prospectively followed up for 4 months. The main demographic, clinical, laboratory, and X-ray findings of AS patients enrolled in the present study were summarized in Table 1. Peripheral involvement was registered in 14 patients (35%). Most of the patients had inactive or moderate disease activity (median ASDAS-CRP: 2.1). The serum concentrations of sclerostin in AS patients and in healthy controls were described in Figure 1. In our group, serum sclerostin levels (median (25th–75th percentile) were significantly higher in healthy controls (18.04 (13.6–24) pg/ml) than in AS patients (6.46 (4.5–11.1) pg/ml; *P* value <0.01) (Figure 1).  Figure 2 shows the receiver operating characteristic (ROC) curve for serum levels of sclerostin in patients with AS. Serum levels ≤ 13 pg/ml have sensitivity and specificity of 82.5 and 90%, respectively, with a likelihood ratio of 8.25. Furthermore, sclerostin serum levels, although without a significant difference, were found to be lower (4.2 (3.1–5.9) pg/ml) in patients with ankylosis of sacroiliac joints (grade IV) than in patients with grade II and III sacroiliitis (5.07 (4.07–6.4) pg/ml, *P* = 0.07). However, no significant correlation was found between serum sclerostin levels and radiographic damage, assessed by mSASSS (Figure 3), and no significant correlations were found between serum sclerostin levels and clinical indices of activity and disability or with laboratory parameters.

**Figure 4:**
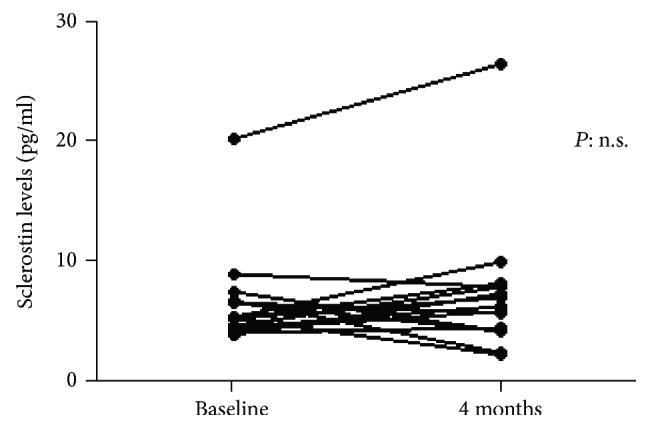
Sclerostin serum levels in patients with AS (*n* = 15) who started an anti-TNF due to high disease activity before (baseline) and after 4 months of treatment.

Sclerostin levels did not show significant changes after 4 months of anti-TNF therapy in the 15 prospectively evaluated patients (Figure 4).

## 4. Discussion

In the present study, we evaluated the possible role as a biomarker of serum level of sclerostin in a cohort of patients affected by AS.

The understanding of AS pathogenesis is a critical issue in order to prevent the bone formation, probably the most important cause of disability and reduced quality of life in AS patients. In the last years, several new players involved in the AS pathogenic mechanisms have been suggested, with encouraging results. In our study, we confirmed the presence of significantly lower serum levels of sclerostin in patients with AS compared to healthy controls. This result is in keeping with the previous studies published in the literature. In particular, Appel et al. showed significantly lower serum sclerostin levels in a cohort of 46 patients with AS compared to healthy subjects and patients with osteoarthritis. Serum sclerostin levels were also significantly associated with the development of new 1-year and 2-year follow-up syndesmophytes, reinforcing the hypothesis that the lower expression of Wnt inhibitors contributes to the activation and differentiation of osteoblastic cells [9]. In the same study, the authors demonstrated a reduced local expression of sclerostin at the level of bone tissue samples obtained from patients with AS [9]. These results were confirmed in other studies evaluating AS patients [22, 23].

In the study conducted by Saad and colleagues, sclerostin levels increased after 6 months and 12 months of anti-TNF treatment. Furthermore, the authors themselves reported persistently reduced sclerostin levels in patients with a high disease activity [22].

In our study, sclerostin levels ≤ 13 pg/ml showed sensitivity and specificity of 82.5 and 90%, respectively. However, this high discriminative capacity works only if compared with healthy controls and further analysis should be performed, in particular, in patients affected by disease belonging to the spondyloarthritis group such as psoriatic arthritis, or in patients with osteoarthritis. In our group, we did not find any correlations with disease activity; this result could suggest that inflammation and sclerostin levels might be unrelated. Furthermore, considering the group of 15 prospectively patients evaluated, no significant differences were found between serum sclerostin levels at baseline and after 4 months of anti-TNF treatment, although there was a tendency towards increasing levels after treatment. According to the study conducted by Saad and collaborators, this could be linked to the short duration of follow-up in our patients, to the small number of patients or to the hypothesis that other cytokines or cellular pathways may play a role in the suppression of sclerostin expression. Recently, a systematic review did not suggest the role of sclerostin as a potential biomarker, due to the lack of significant differences in serum levels of sclerostin between cases and controls [24]. However, several limitations should be considered in this meta-analysis. First, there are very few studies and a relative small number of patients; thus, the limited size might affect the conclusion. Second, the articles, which only support median and range, were excluded [24].

The results of our study are difficult to interpret. We did not find any significant association between sclerostin levels, disease activity, and radiographic damage. This result reinforces the hypothesis that inflammation and new bone formation are paired, but independent processes and sclerostin levels are not consequently influenced by the presence of inflammation, the duration of disease, or disease activity. It can be hypothesized that the pathogenetic processes that cause a decrease in sclerostin levels could occur early and autonomously self-sustain. However, the very low serum sclerostin levels found in AS patients in respect to controls reinforce the hypothesis of its involvement in AS pathogenesis.

## 5. Conclusions

The results of our study suggest a possible role of sclerostin in the identification of AS patients. However, further studies are needed to prove the role of sclerostin as a disease activity biomarker and progression of disease in AS.

## Figures and Tables

**Table 1:** The main demographic clinical and X-ray features of AS patients (*n* = 40).

Male/female	30/10
Age (median/25th–75th percentile) year	50 (40.5–56.75)
Disease duration (median/25th–75th percentile) year	12.5 (6.2–21.5)
HLA-B27, *n* (%)	28 (70)
CRP, mg/dl (median/25th–75th percentile)	0.5 (0.2–0.9)
ESR mm/hr (median/25th–75th percentile)	12.5 (5–20.7)
VAS global health (median/25th–75th percentile)	4.75 (3–5.9)
VAS physician (median/25th–75th percentile)	4 3–5
ASDAS-CRP (median/25th–75th percentile)	2.1 (1.5–3.3)
ASDAS-ESR (median/25th–75th percentile)	2.2 (1.4–3.25)
BASDAI (median/25th–75th percentile)	3.65 (2–5.2)
BASMI (median/25th–75th percentile)	2 (1–5)
BASFI (median/25th–75th percentile)	1.65 (1–3.9)
Sacroileitis IV grade, *n* (%)	14 (35)
Sacroileitis II-III grade, *n* (%)	26 (65)
mSASSS (median/25th–75th percentile)	10 (2.5–29.5)
Peripheral involvement (%)	14 (35)
Enthesitis, *n* (%)	12 (30)
Psoriasis, *n* (%)	3 (7.5)
Inflammatory bowel disease, *n* (%)	5 (12.5)
Uveitis, *n* (%)	9 (22.5)
Treatment, *n* (%)	
NSAIDs	17 (42.5)
DMARDs	6 (15)
Anti-TNF	22 (55)

CRP: C-reactive protein; ESR: erythrocyte sedimentation rate; VAS: visual analogic scale; ASDAS: Ankylosing Spondylitis Disease Activity Score; BASMI: Bath Ankylosing Spondylitis Metrology Index; BASDAI: Bath Ankylosing Spondylitis Disease Activity Index; BASFI: Bath Ankylosing Spondylitis Functional Index; mSASSS: Stoke Ankylosing Spondylitis Spinal Score: DMARDs: disease-modifying antirheumatic drugs; NSAIDs: nonsteroidal anti-inflammatory drugs; TNF: tumor necrosis factor.

## Data Availability

Databases are stored at University of Molise, Department of Medicine and Health Sciences.
